# Nutritional Screening in Hospitalized Patients with Acute Pancreatitis: A Comparative Study in Mexico

**DOI:** 10.3390/jcm15197674

**Published:** 2026-10-04

**Authors:** Mariana Chávez Tostado, Gabino Cervantes Guevara, Francisco Álvarez López, Diana Mercedes Hernández Corona, Alejandro González Ojeda, Clotilde Fuentes Orozco, Miriam Elizabeth Hernández Franco, María Fernanda Isadora Corona Meraz, Milton Omar Guzmán Ornelas, Miriam Méndez del Villar, Tonatiuh González Heredia, Enrique Cervantes Pérez, Guillermo Cervantes Cardona, Verónica Chávez Tostado, Judith Martín del Campo Cervantes

**Affiliations:** 1 Universidad de Guadalajara, Centro Universitario de Ciencias de la Salud, Departamento de Reproducción Humana, Crecimiento y Desarrollo Infantil, Guadalajara 44340, Jalisco, Mexico; miriam.hfranco@academicos.udg.mx; 2 Universidad de Guadalajara, Centro Universitario de Tonalá, Departamento de Ciencias Biomédicas, Centro de Investigación Multidisciplinario en Salud, Doctorado en Investigación Multidisciplinaria en Salud, Tonalá 45425, Jalisco, Mexico; diana.hcorona@academicos.udg.mx (D.M.H.C.); mariaf.corona@academicos.udg.mx (M.F.I.C.M.); milton.guzman@academicos.udg.mx (M.O.G.O.); tonatiuh.gonzalez2923@academicos.udg.mx (T.G.H.); 3 Universidad de Guadalajara, Centro Universitario del Norte, Departamento de Bienestar y Desarrollo Sustentable, Colotlán 46200, Jalisco, Mexico; gabino.cguevara@academicos.udg.mx; 4 Departamento de Gastroenterología, Hospital Civil de Guadalajara “Fray Antonio Alcalde”, Guadalajara 44280, Jalisco, Mexico; francisalvalop@gmail.com; 5 Facultad de Medicina, Universidad de Colima, Colima 28040, Colima, Mexico; agonzalez261@ucol.com; 6 Unidad de Investigación Biomédica 02, Hospital de Especialidades, Centro Médico Nacional de Occidente, Instituto Mexicano del Seguro Social, Guadalajara 44340, Jalisco, Mexico; clotilde.fuentes@gmail.com; 7 Departamento de Medicina Interna, Hospital Civil de Guadalajara “Fray Antonio Alcalde”, Guadalajara 44280, Jalisco, Mexico; enrique.cervantes@academico.udg.mx; 8 Universidad de Guadalajara, Centro Universitario de Ciencias de la Salud, Departamento de Disciplinas Filosofico Metodologicas e Instrumentales, Guadalajara 44340, Jalisco, Mexico; alonso.cervantes@academicos.udg.mx; 9 Departamento de Cirugía, Hospital de Alta Especialidad Tlajomulco de Zúñiga ISSSTE, Tlajomulco de Zúñiga 45640, Jalisco, Mexico; dravro@gmail.com; 10 Departamento de Nutrición, Universidad Autónoma de Aguascalientes, Aguascalientes 20130, Aguascalientes, Mexico; judith.martindelc@edu.uaa.mx

**Keywords:** nutritional screening, acute pancreatitis, nutritional risk

## Abstract

**Background:** Acute pancreatitis (AP) is an acute inflammatory condition with high metabolic demands; however, early detection of nutritional risk is underestimated and there is no universal consensus on which is most effective for predicting nutritional risk. **Objectives:** To compare the level of agreement of nutritional screening methods with subjective global assessment (SGA) in identifying nutritional risk in patients with AP. **Methods:** This study was conducted as a prospective observational cohort in hospitalized patients diagnosed with mild or moderate AP. Screening tools: nutritional risk screening-2002 (NRS-2002), malnutrition universal screening tool (MUST), short nutritional assessment questionnaire (SNAQ), malnutrition screening tool (MST) and clinical assessment via SGA were performed. **Results:** Nutritional risk was found in 55.3% patients via SGA assessment, 43.2% in MST, and 38.6% in NRS-2002 and SNAQ and 10.6% in MUST scales. Patients without nutritional risk exhibited a significantly higher mean BMI than at-risk patients (*p* = 0.026). Patients with moderate AP exhibited a 3.93 increased risk of nutritional risk compared to those with mil AP (*p* = 0.02). The NRS-2002 and SNAQ scales achieved the highest agreement compared with SGA. **Conclusions:** Nutritional risk is highly prevalent in patients with mild and moderate AP, showing a significant increase as clinical severity progresses. The NRS-2002 and SNAQ scales had more agreement and better performance when compared with SGA.

## 1. Introduction

Acute pancreatitis (AP) represents the most frequent gastrointestinal diagnosis among hospitalized patients, accounting for approximately 275,000 admissions and incurring an estimated annual cost of $2.6 billion [1,2]. Furthermore, AP ranks as the fifth leading cause of in-hospital mortality [3]. Globally, AP-related hospitalizations and costs have risen by 20% to 30% over the past decades [2,3]. According to the revised Atlanta classification, AP is categorized as mild (no organ failure and no local or systemic complications), moderately severe (transient organ failure or local/systemic complications without persistent organ failure), or severe (persistent organ failure lasting more than 48 h) [4].

Patients with AP exhibit a typical metabolic pattern of systemic inflammation, hypermetabolism, impaired lipid clearance [5,6] and deranged glucose metabolism [7,8]. This catabolic state accelerates protein breakdown, leading to a net nitrogen deficit that can reach 20 to 40 g/d [8], leading to nutritional insufficiency where approximately 30% of patients are already malnourished at the time of the onset of the disease [9,10], which is associated with higher rates of death, sepsis, severe sepsis, septic shock, and respiratory failure [11]. Despite being largely preventable and reversible, malnutrition frequently remains unrecognized due to limited clinical awareness and the absence of standardized nutritional screening protocols [12]. Implementing nutritional risk screening upon hospital admission is an indispensable first step in clinical care, providing a fast, cost-effective, and standardized approach to detect patients at risk of undernutrition [12]. Rather than establishing a definitive diagnosis, nutritional screening was developed to identify individuals who require further nutritional assessment. According to the 2024 European Society for Clinical Nutrition and Metabolism (ESPEN) guidelines [13], patients with AP should be catalogued as being at moderate to high nutritional risk due to the disease’s catabolic nature and the substantial impact of baseline nutritional status on clinical progression. Consequently, all patients with predicted mild-to-moderate AP should undergo screening using validated instruments such as the Nutritional Risk Screening 2002 (NRS-2002), whereas those with predicted severe AP must automatically be categorized as being at nutritional risk [13]. Although these scoring systems have been validated in hospitalized and critically ill populations, their validity has not yet been established in patients with AP [14].

The British Association for Parenteral and Enteral Nutrition (BAPEN) recommends the Malnutrition Universal Screening Tool (MUST) for nutritional screening in hospital setting [15], whereas the ESPEN recommends the NRS–2002 [16]. The American Society for Parenteral and Enteral Nutrition (ASPEN) also recommends the Patient Generated Subjective Global Assessment (PG–SGA) [17,18] to identify the risk of malnutrition and these are more likely to benefit from early nutritional intervention with improved outcomes [19]. While nutritional screening is widely recommended, their agreement performance has not yet been assessed in patients with mild and moderate AP. To our knowledge, this study provides one of the few studies that compares multiple commonly used screening instruments such as NRS-2002, SNAQ, MST, and MUST against the SGA, specifically within a cohort of mild and moderately AP.

## 2. Materials and Methods

### 2.1. Study Design

This was a cross-sectional analytical study conducted at Civil Hospital de Guadalajara “Fray Antonio Alcalde”, in adult patients admitted to the Gastroenterology Service with a diagnosis of mild or moderate AP between May 2023 and May 2025, to compare the results of the agreement between NRS-2002, MUST, SNAQ, and MST scales compared with SGA for detecting nutritional risk during the first 48 h of hospital admission.

Diagnosis of AP was established within the first 24 h of hospital admission in accordance with international guidelines, which require at least two of the following criteria: (1) characteristic upper abdominal pain, (2) serum amylase or lipase levels > 3 times the upper limit of normal, and (3) characteristic cross-sectional imaging features. AP severity was classified according to the Revised Atlanta Classification (2012) [4]. An initial severity screening was performed upon admission, and the final severity category was confirmed based on the patient’s clinical course during hospitalization. Mild AP was defined by the absence of organ failure and the absence of local or systemic complications. Moderately severe AP was characterized by the presence of transient organ failure (resolving within 48 h) and/or local or systemic complications without persistent organ failure.

Exclusion criteria consisted of underlying end-stage organ failure, pre-existing severe chronic malnutrition or neuromuscular disorders impacting handgrip dynamometry, chronic pancreatitis or pancreatic malignancy, severe cognitive impairment or inability to provide informed consent, pregnancy or breastfeeding, and severe AP. Elimination criteria were incomplete records or voluntary withdrawal.

### 2.2. Sampling Method and Calculation

A non-probability consecutive sampling method was used. All eligible patients admitted during the study period who met the inclusion criteria were consecutively recruited until the required sample size was reached.

The sample size was calculated using the formula for finite populations, based on a target population parameter of *N* = 200 patients obtained from historical institutional admission records. Assuming a 95% confidence level (*Z* = 1.96), an expected proportion of 50% (*p* = 0.50; *q* = 0.50) to maximize sample variability, and a 5% margin of error (*d* = 0.05), the minimum required sample size was determined to be 132 participants, based on the formula output and institutional patient availability.

### 2.3. Data Collection

Eligible patients were identified upon admission. After informed consent, clinical, demographic and laboratory data, including serum albumin, total cholesterol, and lymphocyte count, were obtained from fasting venous blood samples at the time of hospital admission and from medical records. Anthropometric, clinical and functional assessments included age, gender, weight, BMI, and handgrip strength (HGS), severity of AP according to the revised Atlanta classification, length of hospital stay, comorbidities, and clinical complications. Body weight was determined using a TANITA BC 601R scale (Tanita Corporation, Tokyo, Japan). HGS was measured using a Jamar hand dynamometer R (Sammons Preston Inc., Bolingbrook, IL, USA). HGS was defined as the maximally measured grip strength of the dominant hand and was considered reduced for values < 16 kg in females and <27 kg in males [20]. Patients were then followed during hospitalization through electronic medical records to document clinical course, complications and length of stay.

### 2.4. Screening Procedure

All nutritional risk screening tests were performed within 48 h of admission and were performed by the same dietitian. All participants were screened using the NRS-2002, SNAQ, SGA, MUST, and MST scales and were reclassified as “at nutritional risk” if they had a score of ≥3 by the NRS-2002, a score of B or C by SGA, a score ≥ 2 by SNAQ and MST, and ≥1 according to the MUST scales. To minimize evaluation bias and prevent cross-instrument contamination, assessments were administered in a strict, standardized sequence: objective anthropometric and functional measures (height, weight, BMI, and handgrip strength) were recorded first, followed by brief self-reported questionnaires (SNAQ, MST, MUST), the NRS-2002, and finally the reference standard SGA. Each instrument was scored independently on standardized, separated evaluation forms before proceeding to the next tool. The final reference standard SGA classification was calculated and recorded only after all individual screening questionnaires had been completed.

### 2.5. Statistical Analysis

Data were analyzed using descriptive and inferential statistics. Distributional assumptions for all continuous variables were evaluated for normality using the Kolmogorov–Smirnov test. Normally distributed continuous variables were expressed as mean ± standard deviation (SD), while qualitative variables were described with frequencies and percentages. Variables with normal distribution were compared between groups using Student’s two-sample *t*-test and compared using the non-parametric Mann–Whitney *U* test, if abnormal distribution was observed. Associations between qualitative variables were assessed with the Chi-square or Fisher’s exact test. A *p* value < 0.05 was considered statistically significant. The prevalence of nutritional risk was calculated for the total sample and according to the clinical severity of AP. The diagnostic capacity of screening tools was assessed through sensitivity, specificity, positive predictive value (PPV), and negative predictive value (NPV). Cohen’s kappa test (κ) was applied to assess agreement between tools. Crude Odds Ratios (ORs) with 95% confidence intervals were calculated using 2 × 2 contingency tables to estimate the bivariate association between AP severity and nutritional risk. Dynapenia (defined by HGS) was evaluated to assess its relationship with AP severity and nutritional risk status. The statistical analysis was carried out using the Statistical Package for the Social Sciences (SPSS, version 20; IBM Corporation, New York, NY, USA).

## 3. Results

### 3.1. Descriptive Results

The study cohort comprised a total of 132 hospitalized patients. Initially, 185 adult patients with a confirmed diagnosis of AP were screened for eligibility within 24 to 48 h of emergency admission. Of these, 53 patients were excluded prior to enrollment. Specifically, 38 patients met prespecified exclusion criteria: underlying end-stage organ failure (*n* = 14), pre-existing severe chronic malnutrition or neuromuscular disorders affecting handgrip dynamometry (*n* = 11), chronic pancreatitis or pancreatic malignancy (*n* = 8), and severe cognitive impairment or inability to provide informed consent (*n* = 5). An additional 10 patients declined to participate, and 5 patients were excluded due to immediate transfer to the intensive care unit or emergency surgery prior to completing the baseline nutritional assessment. Consequently, 132 patients fulfilled all eligibility criteria, provided written informed consent, and completed the baseline cross-sectional nutritional and functional evaluations.

Among the total cohort of 132 patients, the mean age was 46.8 ± 15.2 years, with a female majority of 61.4% (*n* = 81). In terms of occupational status, 51.5% (*n* = 68) of participants were actively employed, while 33.3% (*n* = 44) were homemakers. Pre-existing clinical comorbidities included type 2 diabetes mellitus in 26.5% of the sample and systemic hypertension in 28.8%. Biochemical parameters upon hospital admission revealed a mean serum glucose concentration of 118.0 mg/dL ± 65.1 mg/dL, a mean serum albumin of 4.7 g/dL ± 1.4 g/dL, and a mean total cholesterol level of 185.0 mg/dL ± 35.5 mg/dL. Anthropometric evaluation demonstrated a mean BMI of 26.4 kg/m^2^ ± 4.7 kg/m^2^, with 43.2% of patients classified as overweight and 24.2% as obese, reflecting a combined excess body weight prevalence of 67.4%. Functional assessment via HGS identified dynapenia (low muscle strength) in 23.5% of the total cohort. Regarding clinical outcomes, the mean length of hospital stay was 3 days ± 4.8 days. Detailed baseline demographic, clinical, and anthropometric data are summarized in Table 1.

### 3.2. Nutritional Risk Prevalence

SGA identified 73 patients (55.3%) as being at nutritional risk. In comparison, screening with MST identified 57 patients (43.2%) at nutritional risk. Both the NRS-2002 and the SNAQ identified 51 patients (38.6%) at nutritional risk. Conversely, the MUST screening tool detected the lowest prevalence of nutritional risk across all instruments, classifying only 14 patients (10.6%) as vulnerable. The comparative prevalence rates of nutritional risk identified by each screening and assessment tool are detailed in Table 2.

Quantitative results demonstrated that according to SGA, patients with nutritional risk compared to the group without nutritional risk were significantly older (40.89 ± 16.31 vs. 35.58 ± 11.18 years; *p* = 0.033) and had a significantly lower BMI (26.54 ± 4.53 vs. 28.62 ± 4.94 kg/m^2^; *p* = 0.026). Also, patients with nutritional risk showed lower serum albumin levels (4.87 ± 1.44 vs. 5.18 ± 1.29 g/dL), lower current weight (70.34 vs. 74.32 kg), and longer hospital stay (4.37 vs. 3.67 days). These results were not statistically significant.

### 3.3. Impact of Pancreatitis Severity on Nutritional Risk

When stratifying nutritional risk by AP severity, the SGA demonstrated a statistically significant difference in risk prevalence between disease severity groups (*p* = 0.003). Among patients with mild AP, 48.0% were classified at nutritional risk, whereas this figure rose markedly to 78.1% in patients with moderate AP.

In contrast, none of the other screening tools demonstrated statistically significant differences in risk detection across disease severity categories. The NRS-2002 identified nutritional risk in 36.0% of mild cases and 46.9% of moderate cases (*p* = 0.27), while the SNAQ yielded nearly identical detection rates, identifying risk in 37.0% of mild cases and 43.8% of moderate cases (*p* = 0.49). Similarly, the MST classified 42.0% of mild cases and 46.9% of moderate cases at nutritional risk (*p* = 0.62). Notably, the MUST recorded the lowest risk identification rates regardless of disease severity, identifying nutritional risk in only 12.0% of mild cases and 6.3% of moderate cases (*p* = 0.35). These comparative findings are detailed in Table 3.

A total of 92 patients (69.7%) were classified as being at overall nutritional risk according to at least one of the screening instruments (SGA, NRS-2002, SNAQ, MST, or MUST). When analyzing the association between AP severity and nutritional risk across the entire patient cohort, results revealed a statistically significant difference (*χ*^2^ = 6.35, *p* = 0.012). While 64.0% of patients with mild AP were categorized as being at nutritional risk across screening tools, this proportion increased to 87.5% in those with moderate AP. Conversely, the absence of nutritional risk was markedly higher in mild presentations (36.0%) compared to moderate cases (12.5%). The bivariate odds ratio observed was 3.93 (95% CI: 1.27–12.12), where patients with moderate AP carry a nearly fourfold increase in the likelihood of nutritional risk compared to those with mild AP. These findings are detailed in Table 4.

### 3.4. Performance Between Nutritional Screening Tools in Agreement with SGA

When evaluating the performance of the four screening tools compared with SGA as the reference standard, the NRS-2002 demonstrated the highest sensitivity at 69.9%, alongside a specificity of 100.0%. The SNAQ had a sensitivity of 65.8% and a specificity of 94.9%. In contrast, the MST and MUST exhibited substantially lower values. Detailed results for all screening tools are summarized in Table 5.

### 3.5. Agreement Between Screening Tools and SGA

The assessment of inter-tool agreement against the SGA reference standard demonstrated that the NRS-2002 achieved the highest level of agreement (*κ* = 0.67), followed closely by the SNAQ (*κ* = 0.58). Both tools exhibited moderate agreement with the clinical reference standard. In contrast, the MST demonstrated only slight agreement (*κ* = 0.19), while the MUST yielded the lowest agreement, showing a result of *κ* = 0.14. Detailed agreement metrics, confidence intervals, and interpretations for all evaluated screening instruments are presented in Table 6.

### 3.6. Clinical Characteristics According to Dynapenia

Within the overall cohort, dynapenia was identified in 23.5% of patients. Overweight status was significantly more prevalent among non-dynapenic individuals than in dynapenic patients (49.5% vs. 22.6%, *p* = 0.008). Conversely, obesity exhibited an inverse trend, with a higher prevalence in the dynapenic group (35.5% vs. 20.8%), although this difference did not reach statistical significance (*p* = 0.095). Regarding disease severity, 71.0% of dynapenic patients presented with mild AP, whereas 29.0% had moderate AP (*p* = 0.477). Overall, 71.0% of dynapenic patients were categorized as being at nutritional risk. When evaluating screening tool performance within the dynapenic subgroup, SGA identified the highest proportion of patients at nutritional risk (64.5%), followed by MST (45.2%), NRS-2002 (35.5%), and SNAQ (29.0%). Conversely, MUST exhibited the lowest prevalence, detecting nutritional risk in only 16.1% of dynapenic patients. Detailed results are shown in Table 7.

## 4. Discussion

AP represents the most frequent gastrointestinal diagnosis among hospitalized patients, accounting for approximately 275,000 admissions and incurring an estimated annual cost of $2.6 billion [1,2]. Furthermore, AP ranks as the fifth leading cause of in-hospital mortality [3]. Globally, AP-related hospitalizations and costs have risen by 20% to 30% over the past decades [2,3]. It is a pathological condition associated with disorders of nutrient assimilation and, as a result, with significant changes in the nutritional status, leading to a profound nutritional insufficiency where approximately 30% of patients are already malnourished at the time of the onset of the disease [9,10]. Because AP triggers a severe hypercatabolic state, nutritional support has become one of the fundamental cornerstones of the clinical management [21]. Consequently, the 2024 ESPEN guidelines [21] recommend that all patients with AP be classified at moderate to high nutritional risk and should be screened using validated screening methods like the NRS-2002 or MUST [13], even though their diagnostic validity has not yet been evaluated specifically in patients with AP. Within the gastrointestinal tract, nutritional risk presents a critical challenge.

One of the most important findings that we observed was that transitioning from a mild to a moderately severe clinical presentation increases the likelihood of nutritional risk nearly fourfold according to the bivariate odds ratio observed of 3.93 (OR = 3.93, 95% CI: 1.27–12.12, *p* = 0.012) from the bivariate cross-tabulation in Table 4. Using SGA reference standard, nutritional risk was present in over half of the overall cohort (55.3%), rising sharply to 87.5% in patients presenting with moderate AP. Also, the evaluation of the level of agreement revealed that NRS-2002 achieved the highest result compared with SGA (k = 0.67, moderate agreement), followed by SNAQ (k = 0.58), whereas MST (k = 0.19) and MUST (k = 0.14) showed slight-to-poor agreement. These findings align with those found in a previous work of our own group, in general gastrointestinal inpatients at the Civil Hospital of Guadalajara “Fray Antonio Alcalde”, where NRS-2002 similarly demonstrated the strongest agreement with SGA (k = 0.53), [22] compared to biomarker-based indices like CONUT (k = 0.36). The consistent superior performance of NRS-2002 across both cohorts highlights the advantage of incorporating acute disease severity parameters alongside weight dynamics in our study. Moreover, despite the high burden of nutritional risk in this cohort, brief screening tools demonstrated marked variability and frequent underperformance in comparison with SGA. Among rapid screening instruments, the NRS-2002 and SNAQ achieved the closest results with the SGA reference standard. NRS-2002 showed the highest overall sensitivity (69.9%) and perfect specificity (100.0%). Conversely, weight-centric instruments like the MUST failed critically, registering a sensitivity of only 17.8% and failing to identify over 80% of patients deemed at risk by SGA. Non-at-risk patients exhibited a significantly higher mean BMI (28.62 ± 4.94 kg/m^2^) than at-risk patients (26.54 ± 4.53 kg/m^2^; *p* = 0.026).

In our exploratory analysis of muscle function, dynapenia was identified in 23.5% of the total cohort. Crucially, dynapenia showed no statistically significant association with AP severity (*p* = 0.477) or overall nutritional risk status (*p* = 0.860). Furthermore, when evaluating tool performances within the dynapenic subgroup, none of the instruments—including SGA (64.5%), MST (45.2%), NRS-2002 (35.5%), SNAQ (29.0%), or MUST (16.1%)—demonstrated significant differential detection rates compared to non-dynapenic individuals. Consequently, standard static screening tools alone may be insufficient to identify patients suffering from mild and moderate AP. Anthropometric analysis also yielded contrasting patterns between overweight and obese individuals in relation to muscle strength. Non-overweight status was significantly associated with a higher prevalence of dynapenia compared to overweight individuals (32.0% vs. 12.3%, *p* = 0.008). Conversely, obesity demonstrated an opposing trend toward a higher rate of dynapenia (35.5% vs. 20.8%, *p* = 0.095). These collective findings carry direct implications for clinical practice in gastroenterology wards. Relying exclusively on rigid, weight-centric screening instruments like MUST creates a false sense of security, leaving a substantial proportion of hypercatabolic patients without timely nutritional support. To optimize clinical management, screening protocols for mild and moderate AP must prioritize instruments that account for disease-induced metabolic stress—specifically the NRS-2002—while routinely incorporating objective functional measures such as HGS. Bridging the gap between metabolic risk screening and functional muscle assessment allows clinicians to initiate early enteral nutrition, preserve lean tissue, and prevent clinical deterioration in patients hospitalized with acute pancreatitis. In future clinical investigations, supplementing initial NRS-2002 screening with a comprehensive assessment like the SGA could enhance screening performance and ensure more timely identification of nutritional risk.

This study has several methodological limitations that should be considered when interpreting our findings. First, the investigation was conducted at a single tertiary care center, which may constrain the generalizability our findings to community-based or secondary healthcare settings with differing diagnostic pathways and demographic profiles. Also, while the SGA was employed as the reference standard in accordance with clinical nutrition guidelines, the SGA itself relies partly on subjective qualitative judgements and history recall, which may introduce misclassification bias in acute inflammatory conditions. Severe AP cases were not included in the study, only mild and moderate AP; severe AP cohorts warrant dedicated investigation. Also, advanced body composition imaging—such as computed tomography (CT) or Dual-Energy X-ray Absorptiometry (DXA)—was not utilized to precisely quantify skeletal muscle wasting and fat distribution, relying instead on functional and anthropometric surrogates. By design, the NRS-2002 explicitly incorporates a severity score corresponding to metabolic stress and disease acute-phase responses; consequently, the higher prevalence of nutritional risk identified by the NRS-2002 in patients with moderate AP is partly explained by tool construction. Moreover, although standardized protocols and separate evaluation forms were implemented to maintain procedural independence, a single trained dietitian administered all screening instruments and the SGA during a single clinical session, creating a potential risk of assessor cross-instrument bias. Future studies utilizing independent, objective biomarkers of catabolism alongside functional parameters will help isolate intrinsic metabolic breakdown from screening tool scoring structures in acute gastrointestinal emergencies.

## 5. Conclusions

Despite the predominance of mild presentations, clinical severity was significantly associated with nutritional risk differences between mild and moderate AP. To optimize clinical management in mild and moderate AP, early nutritional screening should prioritize instruments that account for disease-related metabolic stress and weight dynamics—such as the NRS-2002—paired with other objective functional markers like HGS. Conversely, tools relying on anthropometric metrics or static indicators may underestimate acute nutritional risk in patients with mild and moderate AP.

## Figures and Tables

**Table 1:** Baseline Demographic, Clinical and Anthropometric Characteristics of Hospitalized Adult Patients with Mild and Moderate AP.

Screening Tool	n (%), Mean ± SD
Female Sex	81 (61.4%)
Age (years)	39.2 ± 15.1
Employed	68 (51.5%)
Diabetes Mellitus 2	35 (26.5%)
Systemic Hypertension	38 (28.8%)
Glucose (mg/dL)	118.0 ± 65.1
Albumin (g/dL)	4.7 ± 1.2
Cholesterol (mg/dL)	185 ± 35.5
BMI (kg/m^2^)	26.45 ± 4.7
Overweight	57 (43.2%)
Obesity	32 (24.2%)
Dynapenia	31 (23.5%)
LOS	4.15 ± 4.81

BMI, Body Mass Index; mg/dL, milligrams per deciliter; g/dL, grams per deciliter; kg, kilograms; LOS, length of stay.

**Table 2:** Prevalence of Nutritional Risk Across Screening Instruments and Subjective Global Assessment in Hospitalized Adult Patients with AP.

Screening Tool	No Nutritional Risk n (%)	Nutritional Risk n (%)
SGA	59 (44.7%)	73 (55.3%)
MST	75 (56.8%)	57 (43.2%)
NRS-2002	81 (61.4%)	51 (38.6%)
SNAQ	81 (61.4%)	51 (38.6%)
MUST	118 (89.4%)	14 (10.6%)

SGA, Subjective Global Assessment (Reference Standard); MST, Malnutrition Screening Tool; NRS-2002, Nutritional Risk Screening 2002; SNAQ, Short Nutritional Assessment Questionnaire; MUST, Malnutrition Universal Screening Tool.

**Table 3:** Comparison of Nutritional Risk Detection Across Screening Instruments and Subjective Global Assessment Stratified by AP Clinical Severity.

Screening Tool	Mild AP (n = 100)	Moderate AP (n = 32)	Total	*p* Value
SGA				
No nutritional risk	52 (52.0%)	7 (21.9%)	59 (44.7%)	0.003 *
At nutritional risk	48 (48.0%)	25 (78.1%)	73 (55.3%)	
NRS-2002				
No nutritional risk	64 (64.0%)	17 (53.1%)	81 (61.4%)	0.27
At nutritional risk	36 (36.0%)	15 (46.9%)	51 (38.6%)	
SNAQ				
No nutritional risk	63 (63.0%)	18 (56.3%)	81 (61.4%)	0.49
At nutritional risk	37 (37.0%)	14 (43.8%)	51 (38.6%)	
MST				
No nutritional risk	58 (58.0%)	17 (53.1%)	75 (56.8%)	0.62
At nutritional risk	42 (42.0%)	15 (46.9%)	57 (43.2%)	
MUST				
No nutritional risk	88 (88.0%)	30 (93.8%)	118 (89.4%)	0.35
At nutritional risk	12 (12.0%)	2 (6.3%)	14 (10.6%)	

AP, Acute Pancreatitis; SGA, Subjective Global Assessment (Reference Standard); MST, Malnutrition Screening Tool; NRS-2002, Nutritional Risk Screening 2002; SNAQ, Short Nutritional Assessment Questionnaire; MUST, Malnutrition Universal Screening Tool. (*) Indicates statistical significance (*p* < 0.05).

**Table 4:** Association Between AP Clinical Severity Grade and Nutritional Risk Status in Hospitalized Adult Patients.

AP Severity Grade	No Nutritional Risk n (%)	Nutritional Risk n (%)	*p* Value	OR (95% CI)
Mild AP	36 (36.0%)	64 (64.0%)	*p* = 0.012 *	OR 3.93 (1.27–12.12)
Moderate AP	4 (12.5%)	28 (87.5%)		
Total 40 (30.3%) 92 (69.7%)				

AP: Acute pancreatitis. (*) Indicates statistical significance (*p* < 0.05). Overall nutritional risk was defined as testing positive on at least one of the evaluated screening/assessment tools (SGA, NRS-2002, SNAQ, MST, or MUST).

**Table 5:** Performance Metrics of Nutritional Screening Tools Evaluated Against SGA in Hospitalized Adult Patients with AP.

Screening Tool	Sensitivity (%)	Specificity (%)	PPV (%)	NPV (%)
NRS-2002	69.9%	100.0%	100.0%	72.8%
SNAQ	65.8%	94.9%	94.1%	69.1%
MST	52.1%	67.8%	66.7%	53.3%
MUST	17.8%	98.3%	92.9%	49.2%

SGA, Subjective Global Assessment (Reference Standard); MST, Malnutrition Screening Tool; NRS-2002, Nutritional Risk Screening 2002; SNAQ, Short Nutritional Assessment Questionnaire; MUST, Malnutrition Universal Screening Tool. PPV: Positive Predictive Value; NPV: Negative Predictive Value.

**Table 6:** Agreement Analysis (Cohen’s Kappa Coefficients) Between Nutritional Screening Tools and SGA in Hospitalized Adult Patients with AP.

Screening Tool	Kappa Index (κ)	Interpretation
NRS-2002	0.67	Moderate Agreement
SNAQ	0.58	Moderate Agreement
MST	0.19	Slight Agreement
MUST	0.14	Poor Agreement

CI, Confidence Interval; SGA, Subjective Global Assessment; NRS-2002, Nutritional Risk Screening 2002; SNAQ, Short Nutritional Assessment Questionnaire; MST, Malnutrition Screening Tool; MUST, Malnutrition Universal Screening Tool.

**Table 7:** Comparison of Anthropometric, Clinical Severity, and Nutritional Screening Characteristics Stratified by Dynapenia Status in Hospitalized Adult Patients with AP.

Variable	No Dynapenia (*n* = 101)	Dynapenia (*n* = 31)	Total (*N* = 132)	*p* Value
Overweight				
No	51 (50.5%)	24 (77.4%)	75 (56.8%)	0.008 *
Yes	50 (49.5%)	7 (22.6%)	57 (43.2%)	
Obesity				
No	80 (79.2%)	20 (64.5%)	100 (75.8%)	0.095
Yes	21 (20.8%)	11 (35.5%)	32 (24.2%)	
AP Severity				
Mild AP	78 (77.2%)	22 (71.0%)	100 (75.8%)	0.477
Moderate AP	23 (22.8%)	9 (29.0%)	32 (24.2%)	
Nutritional Risk Status				
No nutritional risk	31 (30.7%)	9 (29.0%)	40 (30.3%)	0.860
Nutritional risk	70 (69.3%)	22 (71.0%)	92 (69.7%)	
SGA				
No nutritional risk	48 (47.5%)	11 (35.5%)	59 (44.7%)	0.23
Nutritional risk	53 (52.5%)	20 (64.5%)	73 (55.3%)	
NRS-2002				
No nutritional risk	61 (60.4%)	20 (64.5%)	81 (61.4%)	0.68
Nutritional risk	40 (39.6%)	11 (35.5%)	51 (38.6%)	
SNAQ				
No nutritional risk	59 (58.4%)	22 (71.0%)	81 (61.4%)	0.20
Nutritional risk	42 (41.6%)	9 (29.0%)	51 (38.6%)	
MST				
No nutritional risk	58 (57.4%)	17 (54.8%)	75 (56.8%)	0.79
Nutritional risk	43 (42.6%)	14 (45.2%)	57 (43.2%)	
MUST				
No nutritional risk	92 (91.1%)	26 (83.9%)	118 (89.4%)	0.25
Nutritional risk	9 (8.9%)	5 (16.1%)	14 (10.6%)	

SGA, Subjective Global Assessment (Reference Standard); MST, Malnutrition Screening Tool; NRS-2002, Nutritional Risk Screening 2002; SNAQ, Short Nutritional Assessment Questionnaire; MUST, Malnutrition Universal Screening Tool. (*) Indicates statistical significance (*p* < 0.05).

## Data Availability

The data supporting the findings of this study are available from the corresponding author upon reasonable request.

## Abbreviations

The following abbreviations are used in this manuscript:

AP	Acute Pancreatitis
SGA	Subjective Global Assessment
MST	Malnutrition Screening Tool
NRS-2002	Nutritional Risk Screening 2002
SNAQ	Short Nutritional Assessment Questionnaire
MUST	Malnutrition Universal Screening Tool
BMI	Body Mass Index
HGS	Handgrip Strength
LOS	Length of Stay
PPV	Positive Predictive Value
NPV	Negative Predictive Value
CI	Confidence Interval
OR	Odds Ratio
SPSS	Statistical Package for the Social Sciences
ESPEN	European Society for Clinical Nutrition and Metabolism
ASPEN	American Society for Parenteral and Enteral Nutrition
BAPEN	British Association for Parenteral and Enteral Nutrition
PG-SGA	Patient-Generated Subjective Global Assessment
DM	Diabetes Mellitus
CT	Computed Tomography
DXA	Dual-Energy X-ray Absorptiometry
kg/m^2^	Kilograms per Square Meter
mg/dL	Milligrams per Deciliter
g/dL	Grams per Deciliter
